# Fasciculations Following COVID-19 Vaccination—A Case Series of Ten Patients

**DOI:** 10.3390/vaccines14060541

**Published:** 2026-06-19

**Authors:** Ameli Breuer, Vanessa Raeder, Helena Franziska Pernice, Fabian Boesl, Harald Prüss, Heinrich Audebert, Katrin Hahn, Christiana Franke

**Affiliations:** 1 Department of Neurology with Experimental Neurology, Charité—Universitätsmedizin Berlin, Corporate Member of Freie Universität Berlin and Humboldt-Universität zu Berlin, 10117 Berlin, Germany; 2 German Center for Neurodegenerative Diseases (DZNE) Berlin, 10117 Berlin, Germany; 3 Center for Stroke Research Berlin, 10117 Berlin, Germany; 4 Berlin Institute of Health (BIH), 10178 Berlin, Germany

**Keywords:** post-COVID-19 condition (PCC), COVID-19 vaccination, post-COVID-19 vaccine syndrome (PCVS), benign fasciculation syndrome, fasciculations, amyotrophic lateral sclerosis

## Abstract

**Introduction:** Vaccination against COVID-19 has been crucial in controlling the pandemic. While side effects are typically mild, rare neurological complications have been reported. This is a case series of ten patients who reported of persistent fasciculations after COVID-19 vaccination. **Methods:** We describe the clinical presentation and diagnostic work-up of ten patients with new-onset fasciculations in temporal proximity to COVID-19 vaccination. Patients with prior SARS-CoV-2 infection or known alternative causes of fasciculations were excluded. Routine clinical data, including neurological examination, laboratory results, and electrophysiology (electromyography and nerve conduction studies), were analyzed. **Results:** Ten patients (5 male, 5 female; mean age 42.4 years) reported fasciculations beginning within 6 h to 13 days post-vaccination and persisting for 2–12 months at the time of presentation. Fasciculations were accompanied by additional symptoms such as paresthesia and fatigue. Laboratory results were mostly unremarkable; two patients had positive myositis antibodies without clinical correlates. Electrophysiology was unremarkable in six patients, while fasciculation potentials were detected in four patients. Nine were diagnosed with probable benign fasciculation syndrome (BFS), and one met diagnostic criteria for amyotrophic lateral sclerosis (ALS). **Discussion:** In this small, retrospective case series, most cases of post-vaccination fasciculations were benign and compatible with BFS. Whether BFS onset was causally linked to vaccination or due to a nocebo effect remains unclear. One patient was diagnosed with ALS, though a causal link remains speculative given the study’s limitations and rarity of similar reports. Larger, prospective studies are needed to validate these observations and explore underlying pathophysiological mechanisms.

## 1. Introduction

The development of vaccines has played a crucial role in the fight against the global Coronavirus Disease 2019 (COVID-19) pandemic caused by Severe Acute Respiratory Syndrome Coronavirus 2 (SARS-CoV-2). Within an unprecedentedly short timeframe, several vaccines were developed and approved, all of which have been shown to safely and effectively decrease SARS-CoV-2 transmission and reduce COVID-19-related mortality. Common vaccine side effects include mild, self-limiting symptoms such as injection-site pain and swelling, fever, myalgia, and headache, which typically resolve within a few days [1]. In rare cases, severe neurological adverse events have been reported, including Guillain-Barré syndrome, acute disseminated encephalomyelitis, acute transverse myelitis, and cerebral venous sinus thrombosis with or without vaccine-induced immune thrombotic thrombocytopenia (VITT) [2,3,4].

Fasciculations are spontaneous, intermittent contractions of muscle fibers that do not result in limb or joint movement. Although a hallmark symptom of amyotrophic lateral sclerosis (ALS), fasciculations may also occur in other neurological conditions, including diseases of the fore-tip of the spinal cord (e.g., progressive spinal muscular atrophies), peripheral neuropathies, chronic denervation due to radiculopathy, and neuromuscular hyperexcitability syndromes. Systemic causes include electrolyte imbalances, metabolic disorders such as thyroid or parathyroid gland disease, and medication side effects (e.g., acetylcholinesterase inhibitors, corticosteroids, and succinylcholine) [5].

Even healthy individuals may occasionally experience fasciculations [6,7]. Benign fasciculation syndrome (BFS) is diagnosed when persistent fasciculations follow a non-progressive course, neurological examination reveals no weakness, muscle atrophy, or pathologically brisk reflexes, other potential causes have been excluded, and needle electromyography (EMG) shows no multifocal or generalized spontaneous activity beyond isolated fasciculation potentials. BFS may be accompanied by sensory disturbances, subjective weakness, and muscle cramps. Because fasciculations are a well-recognized feature of amyotrophic lateral sclerosis (ALS), their occurrence often causes considerable concern among affected individuals, particularly among healthcare professionals. Symptoms may be exacerbated by factors such as physical exercise, stress, fatigue, and caffeine consumption. This phenomenon has occasionally been referred to as fasciculation anxiety syndrome in clinicians (FASICS), reflecting the anxiety that fasciculations may provoke due to concerns about an underlying motor neuron disease [8]. However, prospective studies have yielded heterogenous findings regarding an association between BFS and clinically significant anxiety or depression [9,10,11]. Follow-up studies have shown that BFS does not typically progress to ALS or other severe neuromuscular disorders [9,12,13,14].

Fasciculations following COVID-19 vaccination have only been described in isolated case reports [15,16,17,18]. We report on ten patients who developed persistent fasciculations after COVID-19 vaccination.

## 2. Materials and Methods

Patients reporting of fasciculations in a suspected temporal association with COVID-19 vaccination (i.e., onset between hours up to one month after vaccination) were referred to our university neurology outpatient clinic by general practitioners or neurologists. Patients were interviewed and examined by a neurology specialist between October 2021 and April 2022. All patients had received at least one confirmed COVID-19 vaccination. Patients with confirmed SARS-CoV-2 infection prior to the onset of symptoms or other pre-existing conditions leading to fasciculations were excluded from this report. Laboratory examinations included creatine kinase (CK), myoglobin, myositis antibodies (Mi-2-alpha and -beta, TIF1g, MDA5, NXP2, Ku, PM-Scl 100/75, SPR, Jo-1, PL-7, PL-12, EJ, OJ, SAE and Ro-52), and basic laboratory testing for differential diagnosis of polyneuropathies (differential blood count, erythrocyte sedimentation rate, electrolytes, c-reactive protein (CRP), creatinine, urea, alanine transaminase (ALT), aspartate transaminase (AST), gamma-glutamyltransferase (GGT), thyroid-stimulating hormone (TSH), vitamin B12, HbA1c, blood and urine immunofixation) following diagnostic guidelines of the German Neurological Society [19]. All patients underwent electrophysiological examination, including nerve conduction studies (NCS) and EMG, either in our clinic or prior to their presentation in other Neurology outpatient practices or other Neurology departments. In all patients, a minimum of three muscles and nerves were examined following standard procedures. The Gold Coast criteria were applied for ALS diagnosis [20]. No routine follow-up visits were conducted. All diagnostic procedures were part of the standard assessments in our specialized outpatient clinic.

## 3. Results

Ten patients (five female, five male) aged 28 to 69 years (mean age 42.4 years, standard deviation (SD): 12.8 years) reported fasciculations following COVID-19 vaccination. Table 1 summarizes the clinical details. The latency between vaccination and symptom onset ranged from hours to days (minimum: 6 h, maximum: 13 days, mean 5.4 days, SD: 4.1 days). Symptoms occurred after one (four patients), two (two patients), or three (four patients) vaccine doses. The majority of patients (eight patients) had received a homologous vaccine regimen with the BioNTech-Pfizer (Comirnaty) vaccine. One patient (patient 10) had received a heterologous combination of Moderna (Spikevax) and BioNTech-Pfizer (Comirnaty), and one patient (patient 2) a combination of AstraZeneca (Vaxzevria) and BioNTech-Pfizer (Comirnaty). None had prior COVID-19 infection to their knowledge. Two patients (patients 1 and 6) became infected with SARS-CoV-2 after immunization and after manifestation of the reported symptoms (patient 1: three months after third vaccine dose, patient 6: seven months after third vaccine dose). Patient 1 reported increased fasciculations during the infection, whereas patient 6 did not report any change in symptoms. All other patients tested negative for anti-SARS-CoV-2 nucleocapsid IgG. Two patients (patients 3 and 7) did not have any pre-existing medical history and did not take any regular medication. For all other patients, neither medical history nor medication included conditions (e.g., autoimmune, structural, or metabolic disorders) or medications known to be associated with fasciculations.

**Figure 1:**
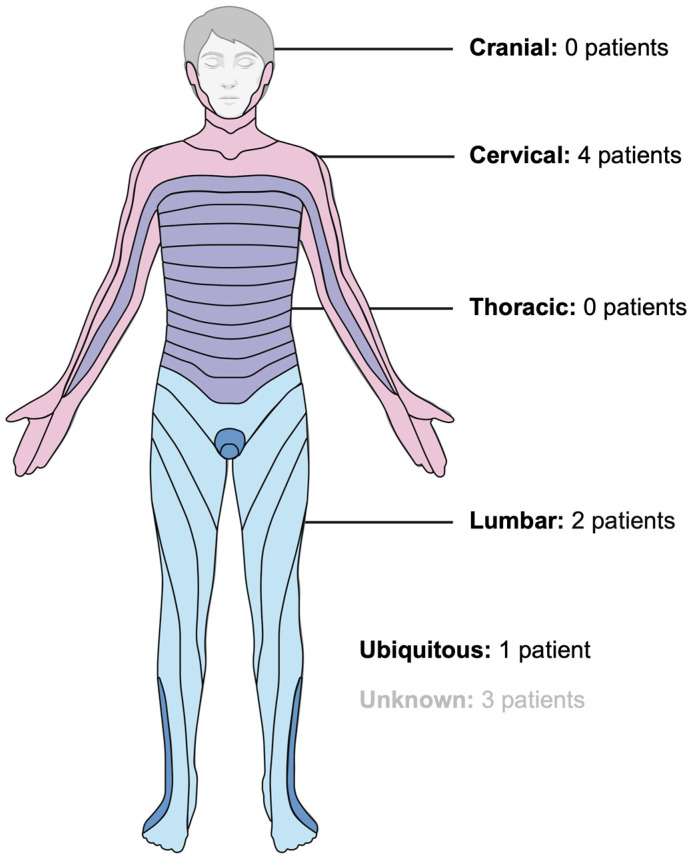
**Myotomal Distribution of Fasciculation Onset.** Number of patients reporting the initial occurrence of fasciculations in the corresponding myotomal regions.

In most cases, fasciculations began in a localized muscle group before spreading to various muscle groups, affecting both upper and lower limbs bilaterally in varying frequency and intensity (Figure 1). Fasciculations persisted between 2 and 12 months (mean: 5.6 months, SD: 3 months) at the time of presentation in our clinic. Patient 3 reported that fasciculations began in the left arm, where the vaccine had been administered. One patient (patient 9) developed right-hand weakness three months after fasciculation onset.

All patients reported additional neurological symptoms, including paresthesia (*n* = 7), fatigue (*n* = 5), neuropathic pain (*n* = 4), limb pain (*n* = 3), myalgia (*n* = 2), subjective cognitive impairment (*n* = 2), visual impairment (*n* = 1), headache (*n* = 1), a unilateral feeling of numbness in the face (*n* = 1), and dizziness (*n* = 1) (see Table 1). Neurological examination was unremarkable in six patients. No patient showed signs of the upper motor neuron, or symptoms indicative of neuromuscular hyperexcitability syndromes (e.g., cramps, spasms, or myotonia). Patient 4 exhibited a limping gait due to a preexisting condition. Patient 5 demonstrated intermittent dystonic posture of the fifth finger on their left hand, resolving upon distraction. Patient 6 reported left-sided facial hypesthesia and hypoalgesia. Patient 9 presented with visible fasciculations in multiple muscle groups, distally pronounced weakness of the right hand, and atrophy of thenar-sided muscles on the right hand. In all other patients, fasciculations were either self-reported or documented through patient-recorded video clips.

Laboratory tests were unremarkable in eight patients. Patients 3 and 4 tested positive for myositis antibodies (patient 3: MDA5 Ab +, PM-Scl100 Ab +, patient 4: PM-Scl75 Ab ++, SAE1 Ab +), without any clinical or electrophysiological correlates for myositis or myopathy, and normal CK and myoglobin levels.

Electrophysiological assessment revealed no pathological findings in six patients. NCS were unremarkable except in one patient (patient 9), where it showed peroneal axonal motor neuropathy. EMG revealed single fasciculation potentials in two patients, while one patient exhibited chronic neurogenic potentials in addition to single fasciculation potentials. Patient 9 displayed ubiquitous fasciculations in the bulbar, cervical, thoracic, and lumbosacral region, accompanied by chronic neurogenic potentials in three regions (see Table 1 for exact description and localization).

In our group of ten patients, nine reported fasciculations without any other clinical abnormalities and were diagnosed with probable BFS. We regard the finding of positive myositis antibodies in two patients as unspecific in the context of their clinical presentation and additional findings. Patient 9 met ALS diagnostic criteria following Gold Coast criteria. For this patient, further diagnostics including spinal magnet resonance imaging (MRI) and a follow-up visit were strongly recommended; however, the patient did not follow up.

## 4. Discussion

This case series provides a detailed clinical characterization of patients reporting fasciculations following COVID-19 vaccination. In nine patients, symptoms appeared to be benign and were considered consistent with BFS, whereas one patient fulfilled diagnostic criteria for ALS.

To date, prior mentions of fasciculations and BFS following COVID-19 vaccination have been limited to single case reports. They include a 48-year-old woman who developed BFS and migraine aura without headache following Comirnaty vaccination [15], a consecutive web-based reporting ten additional self-reported cases of fasciculations after Comirnaty vaccination that was potentially subject to sampling bias [16], a patient with multifaceted post-vaccination symptoms including fasciculations [17], and a 43-year-old male who developed isolated BFS following vaccination with the CoronaVac vaccine [18].

As there is no systematic surveillance of vaccine side effects in Germany, the true incidence of fasciculations following COVID-19 vaccination remains unknown. National pharmacovigilance systems, such as the Paul-Ehrlich-Institut in Germany or the Vaccine Adverse Event Reporting System (VAERS) in the United States, rely largely on voluntary reporting of suspected adverse events and therefore do not allow reliable estimation of symptom incidence. Moreover, transient fasciculations may be perceived as benign and may not prompt affected individuals to seek medical attention, potentially leading to underreporting. Consequently, based on the available data and our findings, it remains unclear, whether BFS onset was merely temporally associated with vaccination, triggered by or causally related to vaccination, or influenced by other factors, including the nocebo effect.

To our knowledge, only one other case of a patient developing ALS after COVID-19 vaccination has been reported so far. This patient, who was relatively young (47 years old) and had a positive family history of ALS, developed left-sided weakness and dysphagia one day after receiving the Janssen COVID-19 vaccine [21]. Similarities may also be drawn to a case of FUS (Fused in Sarcoma)-associated ALS in a 15-year-old girl following human papilloma virus (HPV) vaccination [22]. The absence of additional reports of ALS onset in the context of vaccination suggests that such occurrences are either extremely rare or coincidental.

An interesting observation is that all patients in our group developed symptoms following administration of an mRNA-based vaccine. However, this finding should be interpreted in the context of vaccination practices in Germany during this period: Following the recognition of VITT as a rare but potentially severe complication of the Vaxzevria vaccine, Germany’s Standing Committee on Vaccination (Ständige Impfkommission, STIKO) revised its recommendations in early 2021, resulting in a substantial shift toward the use of mRNA-based vaccines. Consequently, the majority of COVID-19 vaccinations administered in Germany from mid-2021 onwards were mRNA-based [23,24]. Therefore, the exclusive occurrence of cases after mRNA vaccination in our cohort most likely reflects vaccine utilization patterns rather than indicating a vaccine-specific association.

Notably, all patients reported accompanying neurological symptoms resembling PCC [25]. Although some reports suggest that COVID-19 vaccination may induce neurological and other symptoms resembling PCC, data on this phenomenon—sometimes referred to as post-COVID-19 vaccine syndrome (PCVS)—remain limited [26]. Hypothesized mechanisms for vaccine-related neurological symptoms include molecular mimicry, involvement of vaccine adjuvants [27], cross-reactive autoantibodies [28], and persistent spike protein [29], though no robust evidence supports these theories. In a cohort of 50 patients with neurological complaints after vaccination, no pathological findings were identified through standard diagnostic procedures [30]. In contrast, emerging evidence suggests that a substantial proportion of adverse events post-vaccination may be attributable to the nocebo effect [31,32]. In this context, it is noteworthy that approximately half of all suspected PCVS cases worldwide have been reported from Germany [33]. While the reasons for this geographical distribution remain unclear, it may reflect differences in symptom awareness, reporting behavior, healthcare utilization, attitudes toward vaccination, and public attention to the topic rather than true differences in disease occurrence.

We want to emphasize that the benefits of COVID-19 vaccination outweigh potential risks. Severe neurological adverse events remain rare, and the risk of neurological complications from SARS-CoV-2 infection itself is significantly higher [3,34,35].

Limitations of this single-center case series include the small and highly selected patient cohort. We cannot exclude referral bias, as our integrated PCC outpatient clinic has become well known within the Berlin region, potentially attracting patients with similar symptom profiles [25]. Although fasciculations were the leading symptom, the clinical presentation was heterogeneous and accompanied by a broad spectrum of additional neurological complaints, suggesting that fasciculations were often part of a broader symptom complex rather than an isolated manifestation. As fasciculations were only visible in one patient at the time of examination, our findings largely rely on patient’s self-reported symptoms. In this context, the absence of a systematic assessment of psychological factors that might influence symptom perception and reporting, such as health-related anxiety, represents an additional limitation. Furthermore, the retrospective analysis of clinical data partially collected by different examiners, limits direct comparability, particularly with regard to electrophysiological examination. The absence of follow-up visits prevents evaluation of the clinical course or symptom resolution. Finally, as spinal MRI was not performed, we cannot exclude radiculopathies as a potential cause of fasciculations in some cases.

Detailed prospective studies with larger and less selected patient cohorts are needed to validate our observations, and to better characterize the clinical spectrum and potential pathophysiological mechanisms underlying fasciculations after COVID-19 vaccination. Such studies should incorporate standardized neurological, electrophysiological, and psychological assessments beyond routine clinical diagnostics to help distinguish potential biological mechanisms from factors such as the nocebo effect. In addition, retrospective analyses of national and international adverse event reporting systems may help to differentiate background population adverse events from true vaccine-associated adverse events. Such analyses could also be used to explore whether these events cluster within specific vaccine manufacturing lots.

In summary, in this small case series, perceived fasciculations following COVID-19 vaccination were largely benign. Due to the methodological limitations of our study and the currently available evidence, no conclusions can be drawn regarding a potential causal relationship between COVID-19 vaccination and the onset of ALS. Prospective, standardized studies are needed to validate our findings, distinguish correlation from causation, and further explore underlying mechanisms, including the nocebo effect. Additionally, systematic analyses of pharmacovigilance databases may help distinguish background population rates from potentially vaccine-associated events.

## Figures and Tables

**Table 1:** Clinical summary of patients.

Patient	Vaccine 1	Vaccine 2	Vaccine 3	Onset of Symptoms After Vaccine Number	Latency Until Onset of Symptoms	Accompanying Neurological Symptoms	Electromyography
1	Comirnaty	Comirnaty	Comirnaty	3	6 h	Paresthesia, neuropathic pain, cognitive impairment	Unremarkable
2	Comirnaty	Vaxzevria	Comirnaty	3	9 days	Paresthesia, cognitive impairment, visual impairment	Single fasciculation potentials in left vastus lateralis, and right medial gastrocnemius
3	Comirnaty	Comirnaty	-	2	3 days	Myalgia	Single fasciculation potentials in left rectus femoris. Chronic neurogenic potentials in tibialis anterior, and rectus femoris.
4	Comirnaty	-	-	1	4 days	Paresthesia, myalgia, limb pain, fatigue	Unremarkable
5	Comirnaty	-	-	1	1 day	Paresthesia, neuropathic pain, fatigue	Unremarkable
6	Comirnaty	Comirnaty	Comirnaty	3	13 days	Paresthesia, limb pain, neuropathic pain, fatigue, headache, unilateral feeling of numbness in the face	Unremarkable
7	Comirnaty	-	-	1	5 days	Paresthesia, limb pain, neuropathic pain	Unremarkable
8	Comirnaty	-	-	1	8 days	Paresthesia, fatigue	Unremarkable
9	Comirnaty	Comirnaty	-	2	7 days	Fatigue	Frequent fasciculation potentials in right biceps brachii and left deltoid. Moderate fasciculation potentials in right masseter, right genioglossus, and right vastus medialis. Positive sharp waves in right vastus medialis. Chronic neurogenic potentials in right biceps brachii, left deltoid, and right vastus medialis.
10	Comirnaty	Comirnaty	Spikevax	3	7 days	Headache, dizziness	Single fasciculation potentials right interosseus, right medial gastrocnemius and right rectus femoris. Chronic neurogenic potentials right rectus femoris.

## Data Availability

The original data presented in this study are included in the article. Further inquiries can be directed to the corresponding author on reasonable request due to privacy reasons.

## Abbreviations

The following abbreviations are used in this manuscript:

ALS	Amyotrophic Lateral Sclerosis
ALT	Alanine Transaminase
AST	Aspartate Transaminase
BFS	Benign Fasciculation Syndrome
CK	Creatine Kinase
COVID-19	Coronavirus Disease 2019
CRP	C-reactive Protein
EMG	Needle Electromyography
FASICS	Fasciculation Anxiety Syndrome in Clinicians
FUS	Fused in Sarcoma
GGT	Gamma-glutamyltransferase
HPV	Human Papilloma Virus
MRI	Magnetic Resonance Imaging
NCS	Nerve Conduction Studies
PCC	Post-COVID-19 Condition
PCVS	Post-COVID-19 Vaccine Syndrome
SARS-CoV-2	Severe Acute Respiratory Syndrome Coronavirus 2
SD	Standard Deviation
TSH	Thyroid-stimulating Hormone
VITT	Vaccine-induced Immune Thrombotic Thrombocytopenia
